# Genic and Global Functions for Paf1C in Chromatin Modification and Gene Expression in Arabidopsis

**DOI:** 10.1371/journal.pgen.1000077

**Published:** 2008-08-22

**Authors:** Sookyung Oh, Sunchung Park, Steven van Nocker

**Affiliations:** Department of Horticulture, Michigan State University, East Lansing, Michigan, United States of America; Netherlands Cancer Institute, Netherlands

## Abstract

In budding yeast, intragenic histone modification is linked with transcriptional elongation through the conserved regulator Paf1C. To investigate Paf1C-related function in higher eukaryotes, we analyzed the effects of loss of Paf1C on histone H3 density and patterns of H3 methylated at K4, K27, and K36 in Arabidopsis genes, and integrated this with existing gene expression data. Loss of Paf1C did not change global abundance of H3K4me3 or H3K36me2 within chromatin, but instead led to a 3′ shift in the distribution of H3K4me3 and a 5′ shift in the distribution of H3K36me2 within genes. We found that genes regulated by plant Paf1C showed strong enrichment for both H3K4me3 and H3K27me3 and also showed a high degree of tissue-specific expression. At the Paf1C- and PcG-regulated gene *FLC*, transcriptional silencing and loss of H3K4me3 and H3K36me2 were accompanied by expansion of H3K27me3 into the promoter and transcriptional start regions and further enrichment of H3K27me3 within the transcribed region. These results highlight both genic and global functions for plant Paf1C in histone modification and gene expression, and link transcriptional activity with cellular memory.

## Author Summary

In eukaryotes, DNA is packaged with histones and other proteins into a dynamic fabric called chromatin. Specific modifications of histones—including methylation of key lysine residues—provide genetic information that acts synergistically with the DNA code. In yeast, the conserved transcriptional regulator Paf1C is required for marking histone H3 within active genes by methylation of Lysine-4, a modification thought to promote gene activity. In higher eukaryotes, this mechanism is elaborated through Polycomb-Group (PcG), which maintains transcriptional repression through cell divisions and involves methylation of Lysine-27 of H3. In this study, we mapped these and other key H3 modifications throughout the genome of the plant *Arabidopsis thaliana* and evaluated the effects of loss of Paf1C on these modifications and gene expression. We found that Paf1C acts globally to maintain histone modification within genes, but is required for appropriate expression of only a handful of genes. These typically showed a high degree of developmental regulation in both Lysine-4 and Lysine-27 methylation. At the flowering regulator *FLC*, targeted by both Paf1C and PcG, loss of activating (Lysine-4) methylation was accompanied by further accumulation of repressive (Lysine-27) methylation. These results provide a link between transcriptional activity and cellular memory.

## Introduction

Post-translational modification of core histones considerably extends the information potential of the genetic code [1],[2]. Methylation of specific residues within the amino-terminal tail of nucleosomal histone H3, in particular, has been tied to activation or repression of transcription within the associated gene(s). For example, where studied in budding yeast and human, nucleosomes containing H3 trimethylated at lysine-4 (H3K4me3) are globally enriched near the transcriptional start sites (TSS) and 5′ regions of active genes, with the degree of enrichment correlating with gene activity [3]–[6]. In yeast, this pattern is thought to be an outcome of cotranscriptional recruitment of the histone methyltransferase SET1 during the early elongation phase [7],[8]. SET1 and homologous methyltransferases such as Trithorax (Trx) in fruit fly and mixed lineage leukemia 1 (MLL1) in human target nucleosomal H3K4 for methylation as components of larger protein complexes [9]–[11]. However, methylated H3K4 likely serves an instructive and promotive role in transcription as well: methylated H3K4 is required for efficient chromatin remodeling at promoters [12],[13], and potentially enhances interaction with the SET1-related complexes [14]. Thus, H3K4me3 may define a mechanism that reinforces the active state of transcription. Di- and trimethylated H3K36 (H3K36me2/me3) is prevalent within transcribed regions in yeast and human, especially near the 3′ ends [15],[16], reflecting cotranscriptional activity of the H3K36-specific SET2 methyltransferase during elongation [17]. Although localized within active genes, H3K36 methylation probably has an overall negative influence on transcription that is mediated at least in part through recruitment of histone deacetylase activity and consequent maintenance of low acetylation levels [18]–[20]. Repressing histone acetylation within transcribed regions is expected to promote internucleosomal interactions and/or chromatin assembly in the wake of PolII, thus minimizing inappropriate intragenic transcriptional initiation at cryptic sites.

Methylation of H3K27 is an elaboration seen only in higher eukaryotes, where it has been linked with transcriptional repression. Dimethylated H3K27 (H3K27me2) is abundant within heterochromatin, whereas in human and fruit fly, trimethylated H3K27 (H3K27me3) is found in frequent islands scattered throughout euchromatin, with extended domains surrounding Polycomb-Group (PcG) protein binding sites including the Hox loci [21]–[25]. In plants, H3K27me3 marks weakly expressed and/or developmentally silenced genes, including known targets of plant PcG proteins [26]–[29]. H3K27 methylation may repress transcription through several mechanisms, including recruitment of PRC1 in metazoans [21], and in plants, direct binding to LHP1, the homolog of Heterochromatin Protein 1 [28]. The conserved PcG protein Enhancer of zeste [E(z)] and associated proteins, designated Polycomb Repressive Complex 2 (PRC2) mediate methylation of H3K27, thus connecting this modification to the maintenance of gene silencing [30].

The PolII-associating factor 1 complex (Paf1C), minimally composed of Paf1, Ctr9, Cdc73, Rtf1, and Leo1 has an important role in establishing patterns of methylated H3K4 and H3K36 by promoting ubiquitination of histone H2B [31],[32] and linking elongating PolII with SET1 and SET2 [7],[8],[15]. Paf1C also has transcription-related roles potentially independent of its function in histone modification, related to elongation [33], suppression of intragenic initiation [34], poly(A) site selection [35], mRNA polyadenylation [36], and 3′ end formation on nonpolyadenylated PolII-generated transcripts [37].

Components of Paf1C are also conserved in higher eukaryotes. The product of the human HRPT2 gene, parafibromin, shows moderate homology with Cdc73, and interacts with human homologs of Paf1, Ctr9, and Leo1 as well as elongating (Ser-2/Ser-5 phosphorylated) PolII *in vivo* [38]–[40]. The human Paf1C complex (hPAF) also contains hSki8, a protein that physically associates with the exosome, required for 3′–5′ mRNA degradation [40]. Similar to yeast Paf1C, hPAF was localized to transcriptionally active genes, and disruption of hPAF led to global reduction in H3K4me3 levels [40]. Both parafibromin and the human Paf1 homolog are known to be disrupted associated with cancers, although potential mechanisms have not been well described [41],[42]. In fruit fly, homologs of Paf1, Rtf1 (dRtf1) and Cdc73 (*hyrax*) colocalize with transcribing PolII [43],[44], and at least dRtf1 is required to maintain global H3K4me3 in chromatin [45].

In *Arabidopsis thaliana*, the *VERNALIZATION INDEPENDENCE (VIP)* genes; *VIP2* (now called *ELF7*), *VIP4*, *VIP5*, and *VIP6/ELF8* encode proteins closely related to Paf1, Leo1, Rtf1, and Ctr9, respectively [46]–[48], whereas VIP3 shows homology with hSki8. VIP3 physically interacts with VIP4 and VIP6 *in vivo* suggesting that these proteins comprise a complex analogous to Paf1C [48]. The *VIP* genes are required for proper expression of a common subset of genes including *FLC*, a MADS-box gene that represses the transition from vegetative growth to flowering. In *vip* mutants, *FLC* is ectopically silenced, allowing flowering soon after germination. *FLC* has emerged as a plant model for understanding the relationship between histone modifications and gene activity [49]. Activity of *FLC* early in development also requires the SET-domain proteins SDG8/EFS and ATX1, and is associated with methylation of H3K4 and H3K36 within *FLC* chromatin [50]–[52]. Silencing of *FLC* in response to growth in cold temperatures (vernalization) is associated with loss of H3 acetylation and H3K4me3, and concomitant accumulation of H3K27me2/me3, within the *FLC* promoter and transcribed region [27],[53],[54]. The VRN2 protein, related to the PRC2 component Su(z)12, participates in K27 methylation at *FLC* and is required to maintain *FLC* silence in vernalized plants [53],[55],[56].

Unlike specific Paf1C components in yeast, null *vip* mutants (*vip3/4/5/6*) did not exhibit discernible reduction in the amount of H3K4me2/3 or H3K36me2 [48] when assayed at a whole-organism and whole-genome level, indicating that at least the bulk of these modifications is not dependent on Paf1C. However, H3K4me3 levels were reduced within *FLC* chromatin [49]. This loss of H3K4me3 could result indirectly from transcriptional inactivity, or could reveal a locus-specific role for plant Paf1C in mediating H3K4me3 deposition. To investigate potential mechanisms of histone modification and gene regulation modulated by Paf1C-related proteins in plants, and the relationship between Paf1C activity and H3K27 methylation/PcG-associated gene silencing, we mapped histone H3 modifications (trimethylation of H3K4/27, dimethylation of H3K36) and H3 occupancy in the entire Arabidopsis genome from wild-type and *vip3* mutant plants using ChIP combined with high-density tiling microarrays, and linked this information with Paf1C-dependent gene expression.

## Results

### Mapping of Wild-Type and Paf1C-Dependent H3 Occupancy and H3 Modifications in the Arabidopsis Genome

To investigate the influence of plant Paf1C activity on histone modifications in plants, we mapped H3 occupancy and distribution of specific histone H3 methylations at high resolution throughout the genome of both wild-type Arabidopsis plants and mutants homozygous for a null allele of the Paf1C-related gene *VIP3*. We targeted H3K4me3 and H3K36me2 because previous analyses showed that both modifications are associated with transcriptional activity of the flowering regulatory gene *FLC* [47],[51], which is silenced in *vip* mutants [57], and because of the observation that methylation of H3K4 and H3K36 are associated with Paf1C activity in budding yeast [7],[8],[15]. We also analyzed H3K27me3, as this modification was reported to be associated with *FLC* silencing in vernalized plants [27].

**Figure 1:**
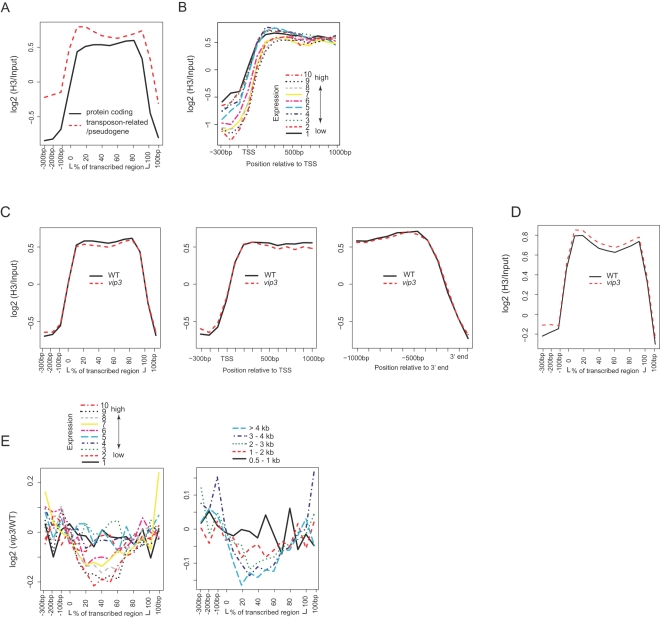
H3 Signal Profiles within Genes and Effects of Paf1C Disruption. (A, B) H3 signal profiles associated with gene type and expression level. (A) Mean genic positional signal for H3 was calculated independently for ∼14,500 likely protein-coding genes and ∼3,000 transposon-related or likely pseudogenes from our ∼18,000 gene set, and is depicted across the promoter regions (shown in bp from −300 to 0 relative to the presumed transcriptional start site), transcribed regions (shown proportionally from 0 to 100% of total length), and 3′ regions (shown in bp from 0 to +100 relative to the presumed 3′ end). (B) Protein-coding genes were sorted into ten-percentile bins according to their expression level, as estimated from publicly available microarray data (see Materials and Methods). The averaged positional signals for H3 within each bin were plotted. Data is shown for absolute positions across the 5′ end including the presumed transcriptional start site (TSS). (C–E) Genic patterns of H3 signals associated with Paf1C activity. (C) In the left column, signals for all 17,771 genes evaluated are depicted for wild-type plants (WT) or *vip3* mutants across the transcriptional unit as described above for Figure 1A. In the center and right columns, data is shown for absolute positions across the 5′ end including the presumed transcriptional start site (TSS) (center), or across the 3′ end (right) of a subset of 6,180 genes with transcribed regions >2 kb in length. (D) Mean positional H3 signals for transposon-related genes and pseudogenes. (E) Genic patterns of Paf1C-dependent H3 signals with respect to expression level and length. Genic positional signals for H3 were averaged separately for protein coding genes within ten-percentile bins according to expression level (left panel), or twenty-percentile bins according to length of transcribed region (right panel) for *vip3* plants relative to wild-type.

We first estimated chromatin occupancy by H3 using an antibody (H3-CT) specific for the carboxyl-terminal domain. Consistent with previous reports of H3 occupancy in Arabidopsis and other species [58]–[60], when mean positional signals were calculated for a set of 17,771 annotated genes from a variety of classes (see Materials and Methods), a pattern of mean H3 signal was evident, characterized by enrichment within transcribed regions concomitant with depletion within both promoter and 3′ regions, relative to the genomic median (Figure 1A). Protein-coding genes generally showed lower signals than transposon-related genes or pseudogenes. Also consistent with previous reports, we found a clear association between estimated transcriptional activity (see Materials and Methods) and H3 signal depletion within the proximal promoter/TSS (Figure 1B).

When mean positional signals were calculated for the 17,771 gene set for *vip3* mutant plants relative to those for wild-type plants, H3 signals were significantly lower (P value<0.0006; Student's t-test and Wilcoxon rank sum test) across the transcribed region (Figure 1C). The ∼3,000 transposon-related and pseudogenes analyzed within this set showed a slight, but insignificant, increase in H3 signals across the transcribed region (Figure 1D). We sorted protein coding genes into ten-percentile bins based on expression level (see Materials and Methods), and analyzed positional signals for *vip3* mutants relative to wild-type plants within each bin. Interestingly, H3 signals were lower in *vip3* plants within transcribed regions for the five highest expression bins, with the two top expression bins showing a significant (P<0.0001) relative loss of H3 (Figure 1E). When Paf1C-dependence of H3 occupancy was considered as a function of gene length, we noted a subtle relationship between gene length and degree of depletion in *vip3* plants relative to wild-type, with the shortest genes analyzed showing little or no relative H3 loss, and genes with transcribed regions >1 kb in length showing significant (P<0.0001) H3 depletion throughout the transcribed region (Figure 1E). Although these effects were slight when analyzed on a genome level, our results suggests plant Paf1C may have transcription-dependent activity in maintaining H3 and/or nucleosomal density, especially in long genes.

**Figure 2:**
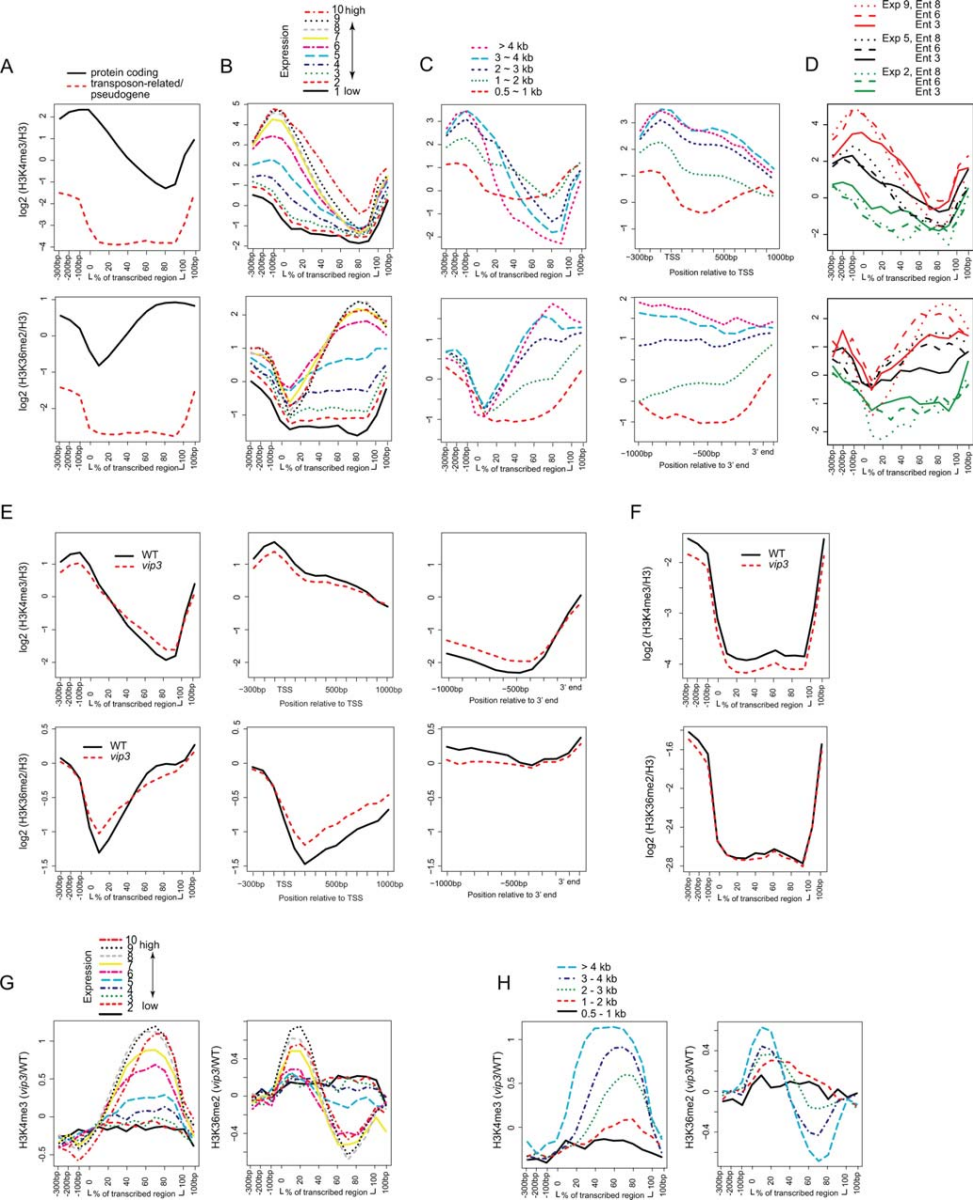
Enrichment of H3K4me3 and H3K36me2 within Genes and Effects of Paf1C Disruption. (A) Mean genic positional enrichment for H3K4me3 or H3K36me2 was calculated independently for protein-coding genes or transposon-related/pseudogenes as described above for Figure 1A. (B) H3K4me3 or H3K36me2 enrichment is depicted for genes within ten-percentile expression level bins as described for Figure 1B. (C) H3K4me3 or H3K36me2 enrichment is depicted for genes within twenty-percentile bins according to length of transcribed region. Enrichment is shown across the transcriptional unit (left panels) or the TSS/5′end (for H3K4me3) or 3′ end (for H3K36me2) (right panels) (D) Protein-coding genes were assigned to ten bins according to tissue-specificity of expression, as estimated by Shannon entropy (see Materials and Methods). Genes in Bin 10 (high entropy) show the most ubiquitous expression across various plant parts, whereas genes in Bin 1 (low entropy) show the most specific expression domains. Mean positional signals were calculated for genes within specific ten-percentile expression (Exp) and entropy (Ent) bins, as indicated. Lines were smoothed using a three-point sliding window. (E) Enrichment for all 17,771 genes evaluated are depicted across the transcriptional unit (left panel), or across the 5′ end/TSS (center) or 3′ end (right) of genes with transcribed regions >2 kb in length, for wild-type plants (WT) or *vip3* mutants. (F) Enrichment within transposon-related genes and pseudogenes. (G) Paf1C-dependent H3K4me3 or H3K36me2 enrichment with respect to expression level, as determined for Figure 1E. (H) Paf1C-dependent enrichment for H3K4me3 or H3K36me2 with respect to gene length, as determined for Figure 1E.

For subsequent analysis of H3K4me3, H3K36me2, or H3K27me3, we expressed signal values relative to total H3 signal. For H3K4me3 and H3K36me2, signals showed a similar pattern at the chromosomal level, being generally above the genomic median in gene-rich chromosome arms and below the genomic median in heterochromatic regions (Figure S1 and data not shown). Considering only protein-coding genes, H3K4me3 increased in the promoter region, peaked near the TSS, and decreased throughout most of the body of the genes (Figure 2A). In contrast, H3K36me2 showed an increase throughout the transcribed regions, peaking near the end of the transcribed regions. Transposon-related/pseudogenes showed depletion of both modifications throughout the extent of the transcribed regions (Figure 2A). Further analysis of genes within expression percentile bins revealed a striking relationship between the enrichment for H3K4me3 or H3K36me2 and transcriptional activity. For example, those genes included in Bin 10, which exhibited the top 10% of expression values, also exhibited the highest peak of H3K4me3 modification near the TSS, whereas those genes with the lowest expression levels showed no obvious peak (Bins 1–3) or only a subtle peak (Bin 4) (Figure 2B). For H3K36me2, genes included in Bins 8 and 9 showed the strongest 3′ peaks, Bin 10 genes showed slightly lower peaks, and genes in Bins 1–4 exhibited low levels of signal throughout the transcriptional unit with no apparent 3′ peak (Figure 2B). Peaks of these two H3 modifications also varied with gene length. When H3K4me3 and H3K36me2 were plotted for genes representing each of five length-assigned bins, the longest genes (Bins 4 and 5, containing genes with transcribed regions >3 kb) displayed the strongest H3K4me3 and H3K36me2 signals at the 5′ and 3′ ends, respectively, whereas Bin 2 (1–2 kb) showed only weak peak signals (Figure 2C). For genes >2 kb in length, the peak of H3K4me3 occurred in a ∼1-kb region at the 5′ end of genes, independent of gene length, whereas the H3K36me2 peak occupied the 3′ ∼one-half of genes, being much broader in longer genes (Figure 2C). These genic patterns and relationship with transcription are similar to those found previously for these modifications in yeast, humans, and where studied, in plants [4],[5],[7],[15],[61],[62] and are consistent with an evolutionarily conserved role for H3K4me3 marking transcriptional engagement, and H3K36me2 as a mark of transcriptional elongation [7],[12],[15].

When signal values were averaged over extended euchromatic or heterochromatic regions, the abundance of H3K4me3 and H3K36me2 was not perturbed in *vip3* plants relative to wild-type (Figure S2 and data not shown). This is consistent with our previous finding that disruption of plant Paf1C did not affect total cellular levels of H3K4me3 or H3K36me2 [48]. However, the effects of loss of Paf1C on distribution of both H3K4me3 and H3K36me2 within genes were substantial: H3K4me3 signals from *vip3* were significantly lower than in wild-type within the peak of this modification near the TSS, but were elevated in the 3′ ∼one-half of the transcribed region (P<2E-16), whereas H3K36me2 signals from *vip3* were significantly lower within the 3′ half, and elevated within the 5′ half of the transcribed region (P<2E-16) (Figure 2E). The most highly expressed genes showed the greatest relative loss of H3K4me3 in 5′ regions, gain in H3K4me3 in 3′ regions, gain of H3K36me2 in 5′ regions, and loss of H3K36me2 in 3′ regions in *vip3* plants (P<0.001 for bins 7–10) (Figure 2G). Transposon-related genes and pseudogenes showed a significant loss of H3K4me3 across the transcribed region (P<1E-16) (Figure 2F).

We also found a substantial length-associated relative increase in H3K4me3 in genes ≥2 kb in length, extending from ∼1 kb downstream of the TSS to the 3′ end (P<2E-06) (Figure 2H). H3K36me2 showed more moderate changes within domains proportional to gene length: an increase within the 5′ ∼one half and decrease within the 3′ ∼one-half of the transcribed region (P<0.005 for genes ≥2 kb in length) (Figure 2H). To analyze this effect independently of any potential relationship between gene length and expression level, we considered differences in relative H3K4me3 or H3K36me2 enrichment within restricted subsets of genes showing similar expression levels. Genes comprising the top 10% expression level bin showed a striking relationship between 3′ H3K4me3 enrichment/H3K36me2 depletion and length (Figure S3). Weakly expressed genes (Expression Level Bin 2) did not show this relationship. We interpret this data as showing that plant Paf1C is required globally not only to maintain a ∼1-kb 5′ peak of H3K4me3 and 3′ enriched region of H3K36me2, respectively, but also for exclusion of these modifications from the remainder of the transcribed region. Because the extent of 5′ H3K4me3 and 3′ H3K36me2 enrichment is generally related to expression level [4],[7],[15], see above, these data imply that the role of Paf1C to maintain appropriate patterns of these modifications is linked to transcriptional activity.

**Figure 3:**
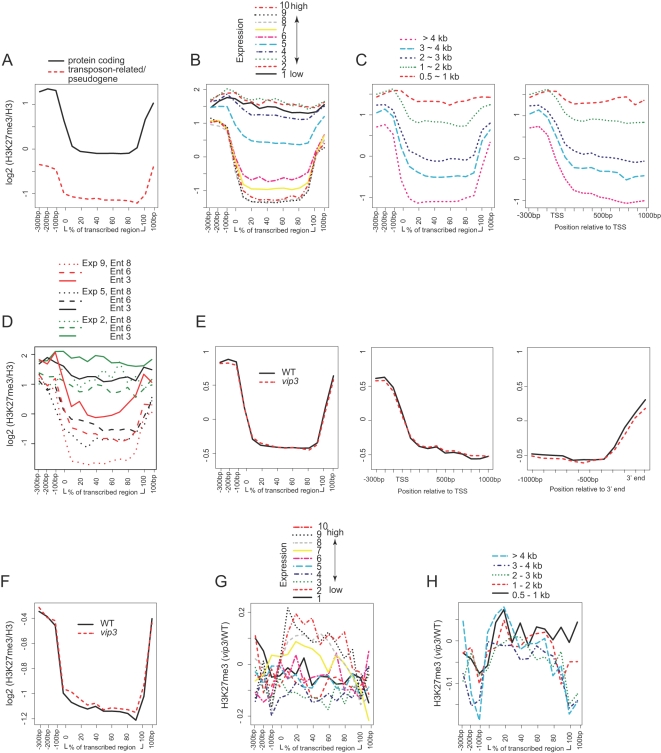
Enrichment of H3K27me3 within Genes and Effects of Paf1C Disruption. (A) Mean genic positional enrichment for H3K27me3 for protein-coding genes or transposon-related/pseudogenes. (B) H3K27me3 enrichment for genes within ten-percentile expression level bins as described for Figure 1B. (C) Mean genic positional enrichment for genes within twenty-percentile bins according to length of transcribed region. (D) Enrichment for H3K27me3 for genes within specific expression level and expression entropy bins as described for Figure 2D. (E) Mean enrichment across the transcriptional unit (left panel) for the ∼18,000-gene set, or across the 5′ end/TSS (center) or 3′ end (right) of genes with transcribed regions >2 kb in length, for wild-type plants (WT) or *vip3* mutants. (F) Enrichment for transposon-related genes and pseudogenes. (G) Paf1C-dependent H3K27me3 enrichment with respect to expression level, as determined for Figure 1E. (H) Paf1C-dependent enrichment for H3K27me3 with respect to gene length, as determined for Figure 1E.

Our analyses of H3K27me3 distribution in wild-type plants support recent reports detailing the global pattern of this modification in Arabidopsis [28],[29],[63]. We found generally strong signals along euchromatic chromosome arms and relatively weak signals within heterochromatic regions (Figure S1 and data not shown) with domains of enrichment occupying ∼8,000 of ∼32,000 annotated Arabidopsis genes (see below), and encompassing the transcribed regions of genes known to be subject to PcG repression, including *FLC* (Figure S4; see below). At the genic level, H3K27me3 was relatively enriched near the TSS and 3′ ends, with weak signals seen across the transcribed region (Figure 3A). Like H3K4me3 and H3K36me2, levels of H3K27me3 were much higher across protein-coding genes than transposon-related and pseudogenes. Similar to the previously reported findings of Zhang et al. [29], and in contrast to our results obtained for H3K4me3 and H3K36me2, H3K27me3 was highest in those genes exhibiting the lowest expression levels (Figure 3B). Analysis of H3K27me3 within gene length bins also revealed a negative relationship between levels of this modification and gene length, with the greatest degree of depletion in the longest genes, and relative enrichment in the shortest genes (Figure 3C). Our results also support the finding of Zhang et al. [29] that genes with low Shannon expression entropy values (tending toward very specific expression patterns) tended to be highly enriched in H3K27me3, whereas genes with high entropy values (widespread expression) generally showed very low H3K27me3 signals (Figures 3D and S5). In contrast, our results do not reveal a strong relationship between entropy and modification by H3K4me3 or H3K36me2 (Figures 2D and S5).

At the chromosomal level, H3K27me3 signals were not noticeably disrupted in *vip3* plants (Figure S2 and data not shown). At the genic level, H3K27me3 signals were essentially unchanged across the transcriptional unit (Figure 3E and data not shown). Highly expressed genes also showed relative gains in H3K27me3 signal within transcribed regions in the *vip3* mutant, but the magnitude of these gains was small compared to the effect seen with H3K4me3 and H3K36me2 (Figure 3G). We saw no obvious gene length-related dependence on Paf1C for H3K27me3 (Figure 3H).

In addition, we examined the relationship between Paf1C-dependence of H3 occupancy or distribution of H3 modifications and tissue-specificity of expression. H3 occupancy was decreased in *vip3* plants within genes showing the highest entropy values (tending toward ubiquitous expression patterns) (Figure S6). This was not unexpected, because genes with highest expression values, which show the greatest depletion of H3, also tend to be ubiquitously expressed (see below). Similarly, entropy-associated profiles of changes in H3 modifications in *vip3* plants were similar to those seen when analyzed for expression level (Figures S6, 2B and 3B). Enrichment for H3K4me3 and H3K36me2, and to a lesser extent H3K27me3, is dependent on expression in subsets of genes with similar entropy values (Figures 2D and 3D). We did not observe a convincing relationship between Paf1C-dependence of H3 occupancy or modification enrichment and entropy when genes within similar expression windows were considered (data not shown). Thus the apparent entropy-associated changes in these modifications dependent on Paf1C may be driven largely by levels of gene expression.

### Mapping of Paf1C-Dependent H3 Modifications within Paf1C-Targeted Genes

**Figure 4:**
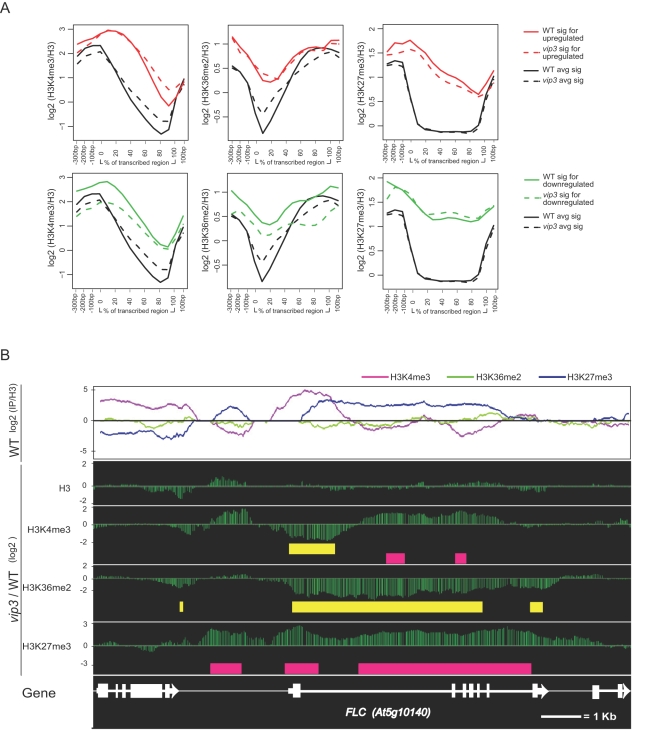
H3 Methylation Profiles and Paf1C-Dependent H3 Methylation Profiles in Paf1C-Targeted Genes. (A) Genic positional signals for H3 lysine methylations as indicated were averaged separately for genes upregulated in *vip3* mutants (top row of panels, red) or downregulated in *vip3* mutants (lower panels, green) for both wild-type plants (solid lines) and *vip3* mutants (dashed lines). Averaged signals for all genes in the protein-coding gene set presented in Figures 2 and 3 are shown in black. (B) Signals for H3 lysine methylations are shown within a ∼14-kb region encompassing the plant Paf1C-dependent gene *FLC* from wild-type (WT) plants (top panel). Lower panels show the relative difference in signals between *vip3* and wild-type chromatin. Horizontal colored bars in these panels indicate regions where significant (2.5-fold change in *vip3*/WT; P<10^−3^ in either *vip3* or WT) differences in signals were observed. A depiction of the *FLC* gene within this region is shown at bottom. Data depicted in this figure were corrected for total H3.

We then considered Paf1C-dependent changes in H3 modifications within those genes whose normal expression depends on Paf1C. Gene expression profiling utilizing microarrays representing most canonical Arabidopsis genes identified a small subset of genes, including the previously identified Paf1C target *FLC*, that were regulated by *VIP3* (data not shown). We observed a statistically significant, average loss of H3K4me3 and H3K36me2 across most of the extent of downregulated genes (Figures 4A and S7). Because enrichment for H3K4me3 and H3K36me2 is correlated with degree of gene expression, this result is consistent with the expected changes in these modifications associated with decreased gene activity. This reveals at least an indirect role for Paf1C in mediating these modifications. For H3K27me3, we observed a slight decrease across upregulated genes, and increase across downregulated genes, but this was not found to be statistically significant (Figures 4A and S7).

Expression of *FLC* is promoted through a mechanism involving plant Paf1C, but is also subject to developmental silencing through a PcG-like mechanism that includes the Su(z)12-like protein VRN2 and accumulation of H3K27me2/3 within the *FLC* gene [27],[53],[56]. To explore the relationship between Paf1C and activating or silencing modifications at *FLC*, we analyzed the chromatin profile of *FLC* in wild-type plants, and the differences in chromatin profiles between wild-type and *vip3* mutant plants. In wild-type plants, H3K4me3 showed a pronounced peak near the TSS and beginning of the first intron (Figure 4B). H3K36me2 showed relatively low levels throughout the *FLC* gene, increasing slightly through the transcribed region and peaking near the 3′ end. Substantial H3K27me3 was seen throughout most of the transcribed region (Figure 4B). In *vip3* mutants, similar to the effect of loss of *VIP3* on the average signal in protein-coding genes, the 5′ peak of H3K4me3 was reduced and levels of H3K4me3 increased in more 3′ regions. H3K36me2 decreased further throughout most of the transcribed region, including the 3′ end (Figure 4B). These observations are consistent with those of Xu et al. [64] who found decreases in both H3K4m3 and H3K36me2 at the 5′ end of *FLC* in plants dysfunctional for the Paf1C-related factor VIP4. In striking contrast, H3K27me3 increased substantially within the proximal promoter, TSS, and the 3′ ∼one-half of the gene (Figure 4B). Thus, at *FLC*, loss of expression is associated with chromatin changes both typical (loss of H3K4me3 and H3K36me2) and atypical (substantial gain of H3K27me3) for Paf1C-regulated genes.

### Chromatin and Expression Characteristics of Paf1C-Targeted Genes

Common distinguishing features of genes dependent on Paf1C for appropriate activity have not been identified. To explore the involvement of chromatin structure in predisposing genes to regulation by Paf1C, we examined the wild-type pattern of H3 modifications among genes that were misexpressed in Paf1C mutants. We found that these genes showed several unique chromatin signatures when compared with the entire set of protein-coding genes. Strikingly, genes misregulated in *vip3* mutants showed much greater enrichment for H3K27me3 across most of the transcribed region (upregulated genes) or the entire transcriptional unit (downregulated genes), relative to average levels for the entire gene set (Figures 4A and S8). These genes were also typified by significantly greater H3K4me3 enrichment throughout much of the transcribed region, with a peak of enrichment 3′ to that seen for the typical gene. Wild-type levels of H3K36me2 were higher throughout the transcribed region, especially near the 5′ end of the transcribed region where levels in the typical gene are lowest. Levels of these three modifications were not dramatically different from the typical gene in the promoter and 3′ regions (Figures 4A and S8). We also analyzed the chromatin signatures of the subsets of gene that we previously found to be misregulated in loss-of-function mutants for the *VIP5* and *VIP6/ELF8* genes, encoding plant homologs of the Paf1C components Rtf1 and Ctr9, respectively [47],[48]. As expected from the observation that the subsets of misregulated genes in *vip5* or *vip6* were largely overlapping with that of *vip3* (data not shown), the chromatin signatures of *VIP5*- or *VIP6*-regulated genes were similarly characterized by a conspicuous enrichment for H3K27me3 across the transcribed region (Figure S9). These genes also showed enhanced H3K4me3 in the 3′ transcribed region, and elevated H3K36me2 in the 5′ transcribed region; this was most apparent for genes downregulated in the mutants (Figure S9).

**Figure 5:**
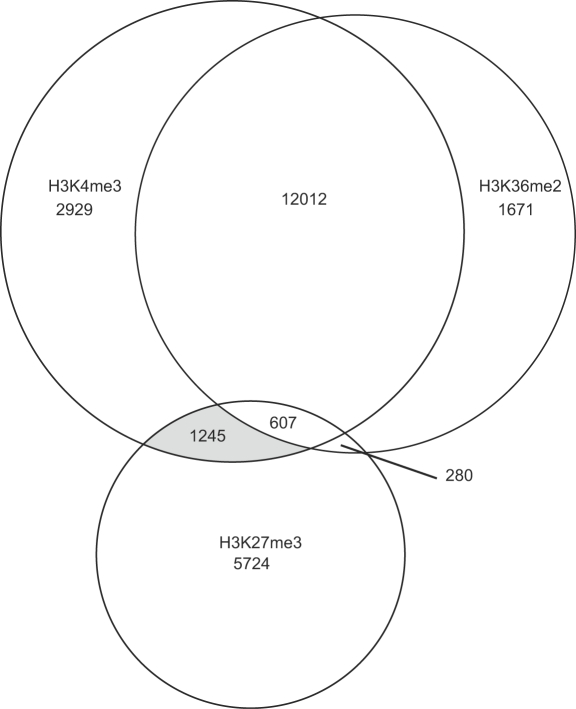
Relationships Among H3 Lysine Methylation Domains. Regions of the genome containing substantial enrichment for H3K4me3, H3K36me2 or H3K27me3 were identified using the TileMap package [82] and linked with genome annotation to identify substantially enriched genes. A Venn diagram indicating the number of annotated genes containing substantial enrichment for single or combinatorial modifications is shown. The shaded area represents the subset of genes enriched in both H3K4me3 and H3K27me3, which shows the most significant overrepresentation for Paf1C-regulated genes.

The finding that Paf1C targeted genes were typically distinguished by combinatorial enrichment for H3K27me3, H3K4me3, and H3K36me2 was intriguing, because we found that H3K4me3, and to a lesser extent H3K36me2, co-occupies only a small subset of genes with H3K27me3 (Figure 5). Indeed, when assigned to groups defined by substantial enrichment for each H3 modification within the transcriptional unit, genes whose expression are positively or negatively influenced by *VIP3* were most significantly overrepresented within a group of genes strongly enriched for both H3K4me3 and H3K27me3 [P value <1E-10 or <1E-05, respectively; Fisher's exact test) (Figure 5 and Table S1). Additionally, when genes were clustered based on distinctions in genic profiles of the modifications as well as enrichment levels, Paf1C-regulated genes were overrepresented in a group of genes showing strong enrichment for H3K4me3 and H3K27me3 and moderate enrichment for H3K36me2, and with H3K4me3 and H3K36me2 distributed broadly across the transcriptional unit rather than in discrete 5′/3′ peaks (Figure S10, Table S2 and data not shown).

**Figure 6:**
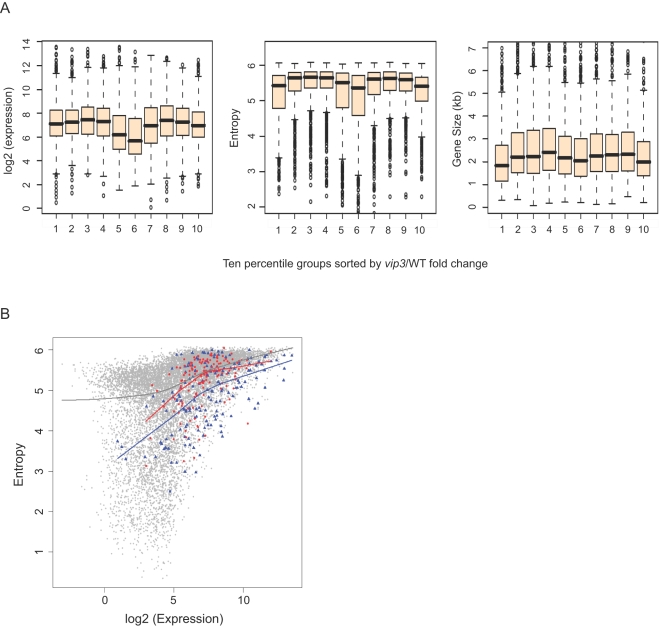
Expression Level, Size and Entropy of Paf1C-Regulated Genes. (A) Box plots showing the distribution of expression level (left panel), expression entropy (middle panel), and gene size (right panel) for ten-percentile subsets of genes according to misregulation in *vip3* mutants. Distribution of genes strongly downregulated in *vip3* relative to wild-type is shown in column 1 of each panel; distribution for genes most strongly upregulated in *vip3* is shown in column 10 of each panel. Colored boxes indicate the 25^th^, 50^th^, and 75^th^ percentiles (bottom, center line, and top of box, respectively). (B) Scatter plot relating gene expression levels with entropy for Arabidopsis genes. Genes strongly upregulated in *vip3* mutants are depicted as red circles, whereas strongly downregulated genes are shown as blue triangles. Locally weighted regression (Lowess) fit lines were superimposed onto the scatterplot (gray, all genes; red, upregulated; blue, downregulated).

High levels of H3K27me3 mark transcriptionally quiescent and developmentally silenced genes, including known targets of plant PcG-like machinery [28],[29]; above. To determine if plant Paf1C has a special role in the regulation of such genes, we compared expression level and entropy of Paf1C-regulated genes with that of the entire gene set. We found that, in wild-type plants, those genes strongly upregulated or downregulated in the Paf1C mutants tended to show low wild-type entropy values, even relative to genes expressed to similar levels (Figure 6 and Figure S9). Scatter plots of expression level and entropy for Paf1C regulated genes, in the context of the entire gene set, clearly showed that within a specific expression level, Paf1C regulated genes tended to show lower entropy values; this was especially significant for genes downregulated in the mutant (P<0.01 and P<0.05 for downregulated or upregulated genes, respectively; Wilcoxon signed-rank test and Student's t-test; see Materials and Methods) (Figures 6B and S9). This is not a trivial result of a potential tissue-specific expression pattern of these genes, as they are expressed relatively ubiquitously [48]; Expression Entropy Bins 8–10 (data not shown). Taken together, these observations suggest that plant Paf1C has an important role in maintaining appropriate expression of developmentally regulated genes.

## Discussion

### Global and Locus-Specific Roles for Plant Paf1C in Chromatin Modifications

In accordance with our earlier report that loss of the plant Paf1C subunits VIP3, VIP4, VIP5, or VIP6 did not affect total cellular levels of H3K4me3 or H3K36me2, here we show that the overall abundance of these modifications within chromatin is not obviously altered in a *vip3* mutant. Instead, loss of plant Paf1C led to redistribution of these modifications within genes: a 3′ shift in the distribution of H3K4me3 and 5′ shift in the distribution of H3K36me2. In yeast, the spatially restricted pattern of genic methyl-K4 and methyl-K36 is thought to depend on the transition from Ser5-phosphorylation to Ser2-phosphorylation within the heptapeptide repeat of the PolII CTD and recruitment of Set1 and Set2, a mechanism that requires Paf1C [65]. If an analogous mechanism linking PolII with these chromatin modifications exists in plants, then a plausible explanation for the general spreading of both H3K4me3 and H3K36me2 within genes in *vip3* mutants is an irregular transition from the Ser-5 to Ser-2 phosphorylated form of PolII. For example, plant Paf1C could be required for interaction of CTD phosphatases with PolII, leading to accumulation of hyperphosphorylated PolII when dysfunctional.

We postulate several scenarios by which this disruption of H3K4me3 and H3K36me2 patterning could directly affect gene activity. Reduction in H3K4me3 near the promoter and TSS may disrupt recruitment of chromatin remodeling machinery needed for efficient initiation, as has been demonstrated for the NURF complex at a Hox promoter [13] or lead to defective pre-mRNA processing [66]. Enhanced levels of H3K4me3 within transcribed regions may promote transcriptional initiation at cryptic sites; the aberrant RNAs thus formed could trigger gene repression through RNAi-related pathways. Loss of H3K36 methylation within transcribed regions may similarly promote aberrant initiation [18]–[20], and RNAi-related silencing, or may alter elongation [15],[67]. Enhanced H3K36 methylation within the 5′/TSS region may disrupt initiation [68],[69].

The apparent global role for Paf1C in maintaining methyl K4/K36 patterns in plants is consistent with observations that budding yeast Paf1C components are abundant and ubiquitously associated with promoters and open reading frames [36],[70]. Interestingly, however, only a small subset of genes are misregulated in yeast or plant Paf1C mutants [35],[48],[71],[72]; this study. What are the distinguishing features of genes whose expression is dependent on plant Paf1C activity? Here, we showed that genes either positively or negatively regulated by plant Paf1C were generally enriched in H3K4me3, H3K27me3, and H3K36me2. We observed some distinction in the degree and pattern of enhanced H3K4me3 and H3K36me2 enrichment between those genes misregulated in *vip3*, and those genes misregulated in *vip5* or *vip6*. This may be attributed to the fact that our gene expression information for *vip5* and *vip6* was archival and derived from plants of a slightly different developmental stage. The observation that both up- and down-regulated genes show similar chromatin profiles may be explained by the fact that some of these genes may be targeted by Paf1C only indirectly. If plant Paf1C primarily targets developmentally regulated genes, then genes positively or negatively regulated by these genes would also be expected to show developmental regulation and accordant chromatin signatures.

The apparent co-occurrence of H3K4me3/H3K36me2 and H3K27me3 domains seen in these genes may result from the net observation of distinct chromatins marked predominantly by H3K4me3/H3K36me2 (in cells where the gene is mostly active) or by H3K27me3 (in cells where the gene is repressed). Another possibility, not mutually exclusive, is that these modifications could be juxtaposed within contiguous chromatin, as seen for H3K4me3 and H3K27me3 in numerous developmentally important genes in mammalian embryonic stem (ES) cells [73]. In ES cells, this so-called H3K4me3/H3K27me3 bivalent domain has been considered as a mechanism to facilitate switching from a repressed to active state, and can resolve to a H3K4me3-dominated or H3K27me3-dominated signature in differentiated cells where the locus is active or repressed, respectively [73],[74].

### Chromatin Dynamics at the *FLC* Gene

The MADS-box gene *FLC* is promoted through a mechanism involving Paf1C during early plant development, and is targeted for repression by a PRC2-like mechanism in response to cold. In wild-type plants, the *FLC* locus showed H3 modification profiles typical for Paf1C-regulated genes: high levels of H3K4me3 at the 5′ end, relatively low enrichment for H3K36me2 at the 3′ end, and a domain of H3K27me3 enrichment throughout much of the transcribed region. However, unlike other genes whose expression is promoted by plant Paf1C, *FLC* exhibited a substantial further accumulation of H3K27me3 when silenced in mutant plants. This suggests a role for Paf1C in antagonizing PcG repression at this locus. Plant Paf1C may also function to antagonize silencing of the several additional known PcG targets, including genes with homeotic functions in flower development, and this could explain the misregulation of these genes and floral abnormalities seen in mutants for various Paf1C-related genes [57].

How might such antagonism be mediated? One of several possibilities is that a role for Paf1C in linking transcription with H3K4/K36 methylation may be elaborated in higher eukaryotes through transcription-associated histone replacement, in which canonical H3 assembled into nascent chromatin is exchanged for ‘variant’ histone H3 (H3.3, also called H3.2 in plants) [75] known to be enriched for methyl-K4 and/or methyl-K36 [76],[77]. Random distribution of H3.3 nucleosomes during replication of active loci would result in a relatively high proportion of methyl-K4-modified H3.3 in nascent chromatin. This content may be further increased during pioneering rounds of transcription through Set1-like H3K4 methyltransferase activity, effectively resetting chromatin to the active state. In contrast, nascent chromatin at silenced loci is expected to be enriched for nucleosomes containing canonical H3, known to be preferentially modified by methyl-H3K27 [76]. H3K27me3 occupancy may be actively reinforced, or passively sustained by modification of canonical H3 in nascent chromatin upon successive replication events. Disrupting Paf1C, and thus the linkage between transcription and H3K4 methylation, would negatively influence resetting of chromatin to the active state and shift the balance of modification to H3K27me3. For some genes, such as *FLC*, even a small disruption of such a balance may then have qualitative effects on chromatin structure and expression.

## Materials and Methods

### Chromatin Immunoprecipitation

Antibodies specific for histone H3 or H3 modifications were as follows: anti-H3-CT (Upstate, Lake Placid, NY; catalog no. 07-690), anti-H3 K4me3 antibody (Abcam, Cambridge, MA; catalog no. ab8580), anti-H3 K36me2 (Upstate 07-369), and anti-H3 K27me3 (Upstate 07-449). The specificity of these anti-H3 K4me3, anti-H3 K36me2, and anti-H3 K27me3 antibodies has been previously documented [78]–[80]. ChIP was carried out essentially as previously described [81], using two grams of aerial tissues from 14-d-old soil-grown plants. Immunoprecipitated DNA fragments were ∼500–600 bp and thus were expected to span two to three nucleosomal units.

Chromatin immunoprecipitation followed by microarray analysis employed the Affymetrix GeneChip Arabidopsis Tiling 1.0R Array (Affymetrix, Santa Clara, CA), as described in the Affymetrix Chromatin Immunoprecipitation Assay Protocol. The Arabidopsis Tiling 1.0R Array represents ∼97% of the Arabidopsis genome with probes spaced every 35 bp. Signal intensities [perfect match (PM)-mismatch (MM)] from two independent biological replicates were quantile-normalized after log_2_-transformation using the TileMap package (http://biogibbs.stanford.edu/jihk/TileMap/index.htm) [82]. Subsequently, for each experiment, signals from immunoprecipitated (IP) or control (input) DNA were linearly scaled to the same mean. We computed a log ratio of the average IP to input value for each probe for further analyses. MvA plots and the correlation values (-R) of two replicates showed high reproducibility (R≥0.979) (Figure S11 and Table S3). To verify enrichments detected by microarray analysis, we carried out standard ChIP followed by semi-quantitative PCR for selected genes (Figure S11 and data not shown).

### Derivation and Analyses of Gene-Level Modification Patterns

For analysis of genic H3 occupancy or H3 modification profiles, we included only those genes spaced 350 bp or greater from an adjacent gene at the 5′ end, and 150 bp or greater from an adjacent gene at the 3′ end. This subset contained 14,485 protein-coding genes and 2,989 transposon-related/pseudogenes, from a total of 31,762 annotated nuclear genes. Gene annotations were taken from release 7 of The Arabidopsis Information Resource (TAIR) genome (ftp://ftp.arabidopsis.org/home/tair/Genes).

Genic profiles were derived by analyzing probe signals for 100-bp windows within the proximal promoter (−350 to −50 relative to the TSS), TSS region (−49 bp to 0 bp to 5% of transcribed region), transcribed region (intervals of 10% of transcribed region from 5% to 95%), 3′ end region (from 95% to 100% of transcribed region to +50 bp relative to the 3′ end), and 3′ flanking region (51 bp to 150 bp relative to the 3′ end). To assess significance of differences in enrichment for H3 (Figure 1) or H3 modifications (Figure 2) within genes between wild-type and *vip3*, we treated positional signals within gene subsets as populations and computed P values using both Student's t-test and Wilcoxon rank sum test.

To identify genomic regions substantially enriched for specific H3 modifications relative to H3 content as described in Figure 5, probe-level t-statistics were computed for each probe based on ChIP vs. input. Neighboring probe signals were integrated by applying a hidden Markov model (HMM) to the probe level statistics with a maximal gap of 1000 bp, a minimal run of 200 bp, and posterior probability cutoff of 0.5. All procedures were performed using the TileMap package [82] and custom Perl scripts. As a result, combined enriched regions for H3K4me3 spanned a total of 18.4 Mbp; for H3K36me2, 19 Mbp; for H3K27me3, 18.2 Mbp. The identified genomic regions were filtered for annotated genes.

### Estimation of Gene Expression Levels and Tissue-Specificity in Wild-Type Plants

AtGenExpress data sets 490, 491 and 492, corresponding to 21-, 22-, and 23-d-old whole plants, respectively (http://www.arabidopsis.org/portals/expression/microarray/ATGenExpress.jsp) were utilized to estimate gene expression levels in wild-type plants. Analysis using data set replicate 475, corresponding to 7-d-old seedlings, gave essentially identical results (data not shown). AtGenExpress data sets 469–547, corresponding to 79 samples representing various tissues and developmental stages, were used to estimate tissue-specificity of expression [83]. We computed Shannon entropy of genes as described [84].

### Analysis of Expression and Chromatin Signatures of Genes Misregulated in *vip3* Mutants

Expression of ∼22,600 genes was analyzed in wild-type and *vip3-1* [57] mutants using the Affymetrix ATH1 GeneChip. Data from CEL files were adjusted for background and normalized using the using the Bioconductor GCRMA package (http://www.bioconductor.org). Statistically significant changes (p<0.001 and 2-fold change) in gene expression between wild type and *vip3* were detected using the Bioconductor LIMMA package [85]. Of 218 upregulated genes and 241 downregulated genes in *vip3* relative to wild type, 139 (upregulated) and 159 (downregulated) were also included in the gene set evaluated for chromatin modifications. To assess statistical significance of differences in chromatin signatures for Paf1C-regulated genes between wild-type and *vip3* mutants, as shown in Figure S7 we computed the 95^th^ percentile confidence intervals for differences in mean positional signals within 1,000 randomly resampled gene sets, each containing 139 (for upregulated) or 159 (for downregulated) genes. To assess the statistical significance of differences in genic chromatin signatures for Paf1C-dependent genes relative to typical genic chromatin signatures as shown in Figure S8, we computed the 95th percentile confidence intervals for the mean positional signals within these gene sets. To assess significance of the lower entropy values observed in genes misregulated in *vip3* mutants, we generated random gene combinations with mean wild-type expression level values similar to those of the upregulated or downregulated gene sets (7.2–7.4 and 7.8–8.0, respectively), and used these populations to compute P values using both Student's t-test and Wilcoxon rank sum test.

### Accession Numbers

Raw data from these experiments has been deposited in the NCBI Gene Expression Omnibus (GEO), accession number GSE7907 (genomic tiling arrays) and GSE10928 (expression arrays).

## Supporting Information

Figure S1 Representative Views of H3 and H3 Lysine Methylation Signal Profiles across Arabidopsis Chromosome IV. Histone H3 and H3 lysine methylation in the Arabidopsis genome were quantified by ChIP combined with microarray analysis (ChIP-on-chip) using antibodies directed against the carboxyl terminus of H3, H3K4me3, H3K36me2, or H3K27me3, and the Affymetrix GeneChip Arabidopsis Tiling 1.0R Array. The entire sequenced region of Chromosome IV is shown above, with numbers indicating the approximate distance (Mbp) from the end of the nonsequenced telomeric rDNA repeats. The centromere is depicted as a blue oval; HK: heterochromatic knob. Raw array data were quantile-normalized and analyzed using Affymetrix Tiling Analysis Software (TAS), and visualized using the Affymetrix Integrated Genome Browser (IGB). For each profile, signals for immunoprecipitate for H3 or each H3 modification are shown relative to the corresponding input signal, and with respect to the genomic median (horizontal colored line). Profiles in the region of the gypsy-class retrotransposon *At4g06591* and an active protein-coding gene, *At4g27760*, are depicted below to illustrate typical genic patterns.
(3.44 MB EPS)

Figure S2 Representative Views of Changes in H3 and H3 Lysine Methylation Signal Profiles across Arabidopsis Chromosome IV Related to Disruption of Paf1C. Data is shown for *vip3* mutants with respect to wild-type plants. Profiles in the region of the gypsy-class retrotransposon, *At4g06566*, and the active protein-coding gene *At4g190202*, *CHROMOMETHYLASE 2*, are depicted below to illustrate typical genic pattern changes.
(3.19 MB EPS)

Figure S3 Relationship Between Paf1C-Dependent, Positional H3 Occupancy, and H3 Methylation Levels and Gene Length for Strongly and Weakly Expressed Genes. Data as analyzed in Figures 1E, 2H, and 3H were filtered to include only those genes in expression bin 10 (blue lines) or expression bin 2 (black lines).
(0.31 MB EPS)

Figure S4 Positional Profiles for H3 Methylation and DNA Methylation within PcG-Regulated Genes in Arabidopsis. ChIP-on-chip was carried out using antibodies directed against H3, H3K4me3, H3K36me2, and H3K27me3. Signals were corrected for H3 and plotted across genomic regions containing *AG*, *AGL19*, *PHE*, and *STM*. A heterochromatic region containing the retrotransposon Ta3 is shown as well. Publicly available information for cytosine methylation is plotted in each frame in parallel with ChIP-on-chip results.
(2.07 MB EPS)

Figure S5 H3 Modifications Related to Gene Expression Level and Entropy. (A) Protein-coding genes were assigned to ten bins according to entropy of expression (see Materials and Methods). The mean positional signals for each modification were calculated independently for each bin. (B) Scores representing the level of each modification within protein-coding genes were calculated (see Materials and Methods), and are illustrated by a color gradient of green (low) to black to red (high) on a plot relating expression level to entropy. Expression level and entropy are indicated on the X and Y axes, respectively. (C) Mean positional signals were calculated independently for genes expressed strongly (top 20% of expression values) in both root and aerial tissues (High, High), strongly only in aerial tissues (High, Low), strongly only in root tissues (Low, High), or expressed to a low level or silenced (lowest 30% of expression values) in both root and aerial tissues (Low, Low).
(6.09 MB EPS)

Figure S6 Genic Patterns of Paf1C-Dependent H3 Occupancy and Methylations with Respect to Tissue-Specificity. Genic positional signals for H3 and H3 lysine methylations as indicated were averaged separately for protein coding genes within ten-percentile bins according to Shannon entropy for vip3 plants relative to wild-type (WT). Data are depicted across the promoter region (shown in bp from −300 to 0 relative to the presumed transcriptional start site), transcribed region (shown proportionally from 0 to 100% of total length), and 3′ region (shown in bp from 0 to +100 relative to the presumed 3′ end).
(0.32 MB EPS)

Figure S7 Significance Analysis of Differences in Chromatin Signatures for Paf1C-Regulated Genes Between Wild-Type and *vip3* Mutants. The 95^th^ percentile confidence intervals were calculated for differences in mean positional signals within 1,000 randomly resampled gene sets for upregulated (left) and downregulated (right) genes.
(0.35 MB EPS)

Figure S8 Significance Analysis of Differences in Genic Chromatin Signatures for Paf1C-Dependent Genes Relative to Typical Genic Chromatin Signatures. The 95^th^ percentile confidence intervals were determined for mean positional signals within 1,000 randomly resampled gene sets for upregulated (left) and downregulated (right) genes.
(0.36 MB EPS)

Figure S9 H3 Methylation Profiles, Expression Level, Size, and Entropy for Genes Misregulated in the *vip5* or *vip6* Mutants. (A) Wild-type genic positional signals for H3 lysine methylations as indicated were averaged separately for those genes upregulated in *vip5* or *vip6* mutants (top row of panels) or downregulated in *vip5* or *vip6* mutants (lower panels). The 95^th^ percentile confidence interval of signals for all genes is depicted with dotted lines. (B) Box plots show the distribution of expression level (left panel), gene size (center panel) and expression entropy (right panel) for ten-percentile subsets of genes according to misregulation in *vip5* or *vip6* mutants. Distribution of genes strongly downregulated in *vip5* or *vip6* relative to wild-type is shown in column 1 of each panel; distribution for genes most strongly upregulated in *vip5* is shown in column 10 of each panel. Colored boxes indicate the 25^th^, 50^th^, and 75^th^ percentiles (bottom, center line, and top of box, respectively). (C) Scatter plot relating gene expression levels with entropy for Arabidopsis genes. Genes strongly upregulated in *vip5* or *vip6* mutants are depicted as red circles, whereas strongly downregulated genes are shown as blue triangles. Lowess fit lines were superimposed onto the scatterplot (gray, all genes; red, upregulated; blue, downregulated).
(1.71 MB EPS)

Figure S10 Clustering and Analyses of Genes According to Chromatin Modification Profile Class. (A) Cluster analyses were performed for the ∼18,000-gene set based on genic positional signals for H3K4me3, H3K36me2, H3K27me3, H3, and DNA methylation. Data was plotted across promoter regions (columns 1–3 in each modification panel), TSS (column 4), transcribed regions (columns 5–14) and 3′ end (column 15). (B) Averaged positional profiles for H3, H3 modifications and DNA methylation [84] are shown separately for each of the four groups. Positions are relative to a representative transcriptional unit shown at bottom. (C) Box plots showing the percentile level of expression (top), expression entropy (middle) and gene length (bottom) for each group. Boxes indicate the 25^th^, 50^th^, and 75^th^ percentiles (bottom, center line, and top of box, respectively).
(1.94 MB EPS)

Figure S11 Reproducibility and Confirmation of ChIP-on-Chip Data. (A) An M versus A (MvA) plot representing signal intensities from the two biological replicates of each genotype is shown. The X-axis is defined as the average of the log base 2 of the intensities from the two replicates, and the Y-axis is the difference of the log base 2 of the intensities from the two replicates. The color bar at right indicates the number of probes on the plots. (B) ChIP analysis of H3 and H3 modifications within the FLC locus is shown. ChIP was carried out using antibodies recognizing H3K4me3 (upper left), H3K36me2 (upper right), H3K27me3 (lower left), and the H3 carboxyl terminus (lower right) within a promoter segment (red), 5′ region (yellow), or 3′ region (green). A 5′ and 3′ region of the ACTIN7 gene was used for an internal control. Band intensities from gel images were quantified and normalized based on those for ACTIN7 (lower band in each gel image). ChIP analysis was performed twice using biologically independent samples and yielded essentially identical results.
(3.96 MB EPS)

Table S1 Representation of *VIP3*-Dependent Genes within Chromatin Enrichment Groups Depicted in Figure 5.
(0.03 MB DOC)

Table S2 Representation of *VIP3*-Dependent Genes within Chromatin Enrichment/Profile Groups Depicted in Figure S10.
(0.03 MB DOC)

Table S3 Reproducibility of Tiling Microarray Results (Correlation Coefficient, R).
(0.03 MB DOC)
