# Supplementary material for: Genic and Global Functions for Paf1C in Chromatin Modification and Gene Expression in Arabidopsis
Source: PLoS Genet. 2008 Aug 22;4(8):e1000077. doi: 10.1371/journal.pgen.1000077 (PMC2515192; doi:10.1371/journal.pgen.1000077)
Supplement: Table S1 — Representation of VIP3-Dependent Genes within Chromatin Enrichment Groups Depicted in Figure 5. (0.03 MB DOC) [file pgen.1000077.s012.doc]

**Table S1. Representation of *VIP3*-Dependent Genes within Chromatin Enrichment Groups Depicted in Figure 5.**

| **Group**  **(No. Genes)** | **^1^*VIP3*-Dependent** | | | | | |
| --- | --- | --- | --- | --- | --- | --- |
|  | **Positive** | | | **Negative** | | |
|  | **No.** | **%** | **P value** | **No.** | **%** | **P value** |
| H3K4me3/ H3K36me2 (12012) | **70** | **0.6** | **(2.3E-08)** | **66** | **0.5** | **(8.3E-06)** |
| H3K4me3 only (2929) | 40 | 1.4 | 1.3E-02 | 23 | 0.8 | 1 |
| H3K36me2 only (1671) | 11 | 0.7 | 2.9E-01 | 17 | 1.0 | 3.2E-01 |
| H3K27me3 only (5724) | 51 | 0.9 | 8.1E-01 | 42 | 0.7 | 5.0E-01 |
| H3K4me3/ H3K27me3 (1245) | **39** | **3.1** | **2.2E-11** | **26** | **2.1** | **9.2E-06** |
| H3K36me2/ H3K27me3 (280) | 2 | 0.7 | 1 | **8** | **2.9** | **2.1E-03** |
| H3K4me3/ H3K36me2/ H3K27me3 (607) | **14** | **2.3** | **1.6E-03** | **16** | **2.6** | **3.7E-05** |
| Entire gene set (24468) | 227 | 0.9 |  | 198 | 0.8 |  |

^1^Indicates number of genes in each group that are downregulated or upregulated in *vip3* mutants relative to wild-type (positive or negative, respectively) and their representation (percentage of all genes) within each group. Numbers shown in bold indicate significant deviation (P value < 0.005; Fisher's Exact Test) from the expected number of such genes in each group; P values shown in parentheses indicate significantly lower representation than expected.
