# Supplementary material for: Genic and Global Functions for Paf1C in Chromatin Modification and Gene Expression in Arabidopsis
Source: PLoS Genet. 2008 Aug 22;4(8):e1000077. doi: 10.1371/journal.pgen.1000077 (PMC2515192; doi:10.1371/journal.pgen.1000077)
Supplement: Table S2 — Representation of VIP3-Dependent Genes within Chromatin Enrichment/Profile Groups Depicted in Figure S10. (0.03 MB DOC) [file pgen.1000077.s013.doc]

**Table S2. Representation of *VIP3*-Dependent Genes within Chromatin Enrichment/Profile Groups Depicted in Figure S10.**

| Group  (No. genes) | **^1^*VIP3*-Dependent** | | | | | |
| --- | --- | --- | --- | --- | --- | --- |
|  | **Positive** | | | **Negative** | | |
|  | **No.** | **%** | **P value** | **No.** | **%** | **P value** |
| 1  (5380) | 44 | 0.82 | 5.44E-01  *5.14E-03* | 44 | 0.82 | 7.12E-01  *1.14E-01* |
| 2  (4086) | **83** | **2.03** | **9.74E-16**  ***9.41E-10*** | **66** | **1.62** | **2.04E-10**  ***4.69E-06*** |
| 3  (4498) | 31 | 0.69 | 9.90E-02  *5.67E-04* | 28 | 0.62 | 1.17E-01  *2.39E-03* |
| 4  (3807) | **1** | **0.03** | **(1.52E-15)** | **1** | **0.03** | **(1.37E-13)** |
| Entire gene set  (17771) | 159 |  |  | 139 |  |  |

^1^Indicates number of genes in each group that are downregulated or upregulated in *vip3* mutants relative to wild-type (positive or negative, respectively) and their representation (percentage) within each group. Numbers shown in bold indicate significant deviation (P value < 5.00 E-06; Fisher's Exact Test) from the expected number of such genes in each group; P values shown in parentheses indicate significantly lower representation than expected. Numbers in italics indicate values when Group 4, which is composed mostly of transposon-related genes and pseudogenes, is not considered.
