# Supplementary material for: Genic and Global Functions for Paf1C in Chromatin Modification and Gene Expression in Arabidopsis
Source: PLoS Genet. 2008 Aug 22;4(8):e1000077. doi: 10.1371/journal.pgen.1000077 (PMC2515192; doi:10.1371/journal.pgen.1000077)
Supplement: Table S3 — Reproducibility of Tiling Microarray Results (Correlation Coefficient, R). (0.03 MB DOC) [file pgen.1000077.s014.doc]

**Table S3. Reproducibility of Tiling Microarray Results (Correlation Coefficient, R)**

|  | **WT-I vs. WT-II** | ***vip3*-I vs. *vip3*-II** |
| --- | --- | --- |
| Input | 0.986 | 0.986 |
| **H3K4me3** | 0.989 | 0.979 |
| **H3K36me2** | 0.980 | 0.983 |
| **H3K27me3** | 0.979 | 0.987 |
| **H3 (H3-CT)** | 0.988 | 0.991 |
